# Phenazinium methyl sulfate

**DOI:** 10.1107/S1600536812026025

**Published:** 2012-06-13

**Authors:** Nai-Qiang Zhang, Ping Li, Jian Dong, Hong-Yu Chen

**Affiliations:** a School of Basic Medical Sciences, TaiShan Medical University, Tai’an 271016, People’s Republic of China; b Faculty of Chemistry and Chemical Engineering, TaiShan Medical University, Tai’an 271016, People’s Republic of China

## Abstract

The title salt, C_12_H_9_N_2_^+^·CH_3_O_4_S^−^, contains an almost planar phenazinium cation [largest deviation from the least-squares plane = 0.040 (3) Å] and a methyl sulfate anion. The sulfate moiety of the latter is disordered over two sets of sites in a 0.853 (5):0.147 (5) ratio. In the crystal, the cations and anions are arranged alternately in layers parallel to (010). The cations pack along [100] with a tilt angle of 28.96 (4)° between this axis and the mean plane and are linked through inter­planar π–π inter­actions [shortest inter­planar distance = 3.421 (4) Å]. N—H⋯O hydrogen-bonding between the cations and anions is also observed.

## Related literature

For background to the use of phenazine in crystal engineering, see: Laursen & Nielsen (2004 ▶). For a related structure, see: Meszko *et al.* (2002 ▶).
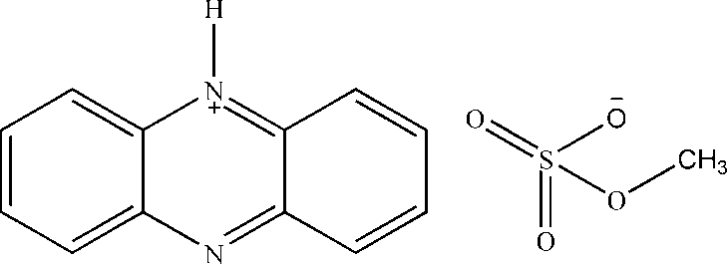


## Experimental

### 

#### Crystal data

C_12_H_9_N_2_^+^·CH_3_O_4_S^−^
*M* *_r_* = 292.31
Triclinic, 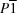

*a* = 5.818 (5) Å
*b* = 9.667 (5) Å
*c* = 11.460 (5) Å
α = 95.241 (5)°
β = 90.336 (5)°
γ = 93.691 (5)°
*V* = 640.5 (7) Å^3^
*Z* = 2
Mo *K*α radiation
μ = 0.27 mm^−1^
*T* = 293 K
0.18 × 0.15 × 0.12 mm

#### Data collection

Bruker APEXII CCD diffractometer
Absorption correction: multi-scan (*SADABS*; Bruker, 2005 ▶) *T*_min_ = 0.951, *T*_max_ = 0.965
3642 measured reflections
2572 independent reflections
2266 reflections with *I* > 2σ(*I*)
*R*_int_ = 0.127

#### Refinement

*R*[*F* ^2^ > 2σ(*F* ^2^)] = 0.066
*wR*(*F* ^2^) = 0.185
*S* = 1.07
2572 reflections
191 parameters
H-atom parameters constrained
Δρ_max_ = 0.54 e Å^−3^
Δρ_min_ = −0.72 e Å^−3^

### 

Data collection: *APEX2* (Bruker, 2005 ▶); cell refinement: *SAINT* (Bruker, 2005 ▶); data reduction: *SAINT*; program(s) used to solve structure: *SIR97* (Altomare *et al.*, 1999 ▶); program(s) used to refine structure: *SHELXL97* (Sheldrick, 2008 ▶); molecular graphics: *XP* in *SHELXTL* (Sheldrick, 2008 ▶); software used to prepare material for publication: *publCIF* (Westrip, 2010 ▶).

## Supplementary Material

Crystal structure: contains datablock(s) global, I. DOI: 10.1107/S1600536812026025/wm2637sup1.cif

Structure factors: contains datablock(s) I. DOI: 10.1107/S1600536812026025/wm2637Isup2.hkl

Additional supplementary materials:  crystallographic information; 3D view; checkCIF report

## Figures and Tables

**Table 1:** Hydrogen-bond geometry (Å, °)

*D*—H⋯*A*	*D*—H	H⋯*A*	*D*⋯*A*	*D*—H⋯*A*
N1—H101⋯O2^i^	0.86	1.82	2.647 (5)	161

Symmetry code: (i) 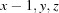.

**Fig. 1.**
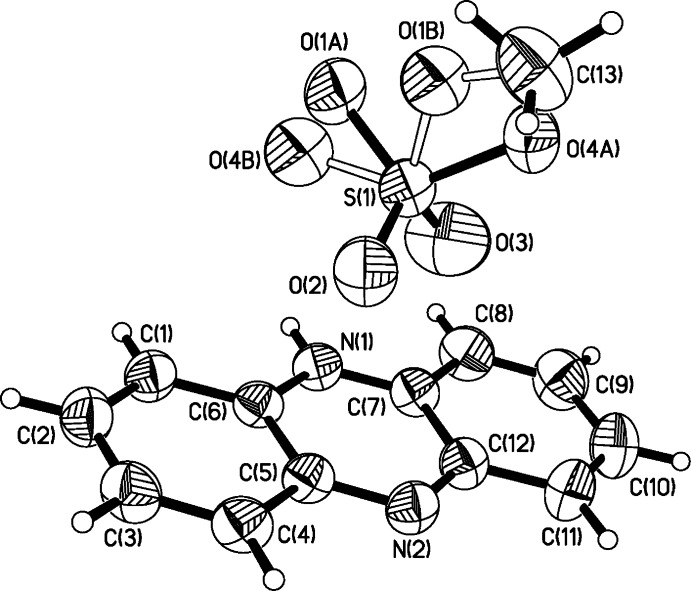
Molecular structure of the title compound with displacement ellipsoids at the 50% probability level. The disorder of the anion is shown.

**Fig. 2.**
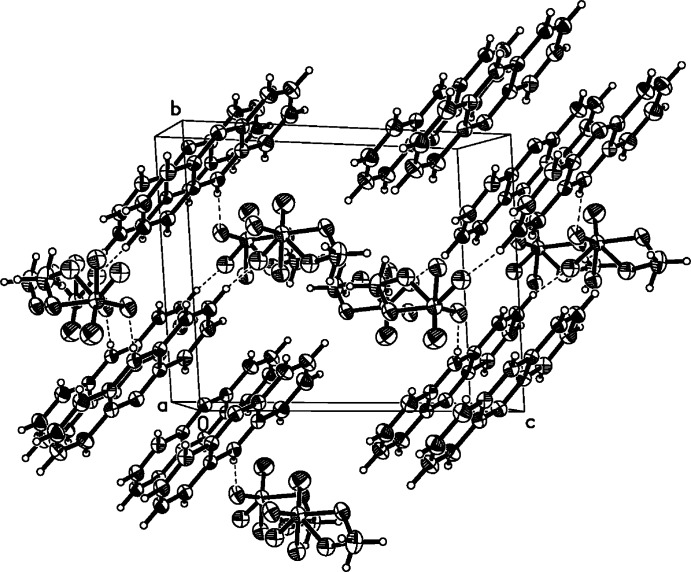
A packing diagram of the title structure viewed approximately along [100]. Hydrogen bonding interactions are shown with dashed lines.

## supplementary crystallographic information

### Comment

In the past decade, phenazines have been widely used as a template in crystal engineering for its two equivalent strong proton acceptors (*sp*^2^ N atoms) and potential weak C—H donor functions, where the aromatic system can act as a good π-donor. Accordingly, phenazine has been employed in the design of charge-transfer complexes and hydrogen bonded assemblies (Laursen *et al.*, 2004). Here, we report the crystal structure of an 1:1 complex of phenazine with methyl sulfate.

The asymmetric unit of the title salt, [C_12_H_9_N_2_]^+^ [CH_3_O_4_S]^-^, contains a phenazinium cation and a methyl sulfate anion (Fig. 1), which is located around the inversion centre. The phenazinium cations show an almost planar configuration, where the largest deviation from the least-square-plane of phenazine is 0.040 (3)Å for C3. The methyl sulfate anions are disordered over two positions in a ratio of 0.853 (5):0.147 (5). The distribution of S—O bond lengths in the methyl sulfate anion is similar to that in the crystal structure of 10-methylacridinium methyl sulfate (Meszko *et al.*, 2002). The S—O bond lengths associated with the methyl group [1.614 (2) Å for the major and 1.486 (18) for the minor part] are longer than the other S—O bonds (1.421 (3) Å, 1.448 (2) Å and 1.423 (2) Å (major part); 1.516 (16) Å (minor part)).

The cations pack along [100] with a tilt angle between the phenazinium plane and the *a* axis being 28.96 (4)°. The shortest plane-to-plane π—π interactions are 3.421 (4) Å. The phenazinium cations and the methyl sulfate anions are alternately arranged parallel to (010) (Fig. 2). Except for Coulombic interactions, there are classical hydrogen bonding interactions between the phenazinium cations and methyl sulfate anions (Table 1), which also play an important role in the stabilisation of the title structure.

### Experimental

To a solution containing phenzine (1.0 g, 0.0056 mmol) in *n*-butyl acetate (20 mL) was added dimethyl sulfate (5.4 mL, 0.057 mmol). The resulting mixture was continuously stired at 373 K for 1 h, then the orange reaction solution was cooled to 283 K. The precipitated yellow solid were collected and recrystallized in ethanol.

### Refinement

All H atoms were geometrically fixed and allowed to ride on their attached atoms, whit C—H = 0.93 Å and *U*_iso_(H)= 1.2*U*_eq_(C) for all phenzine H atoms, and C—H = 0.96 Å and *U*_iso_(H)= 1.5*U*_eq_(C) for the methyl group. The proton attached to the phenazine N atom was also geometrically fixed, with N—H = 0.86Å and *U*_iso_(H)= 1.2*U*_eq_(N). The sulfate part of the anion was modelled as disordered over two sets of sites in a 0.853 (5):0.147 (5) ratio; O atoms of the minor component were refined with isotropic displacement parameters.

### Crystal data

**Table:**

C_12_H_9_N_2_^+^·CH_3_O_4_S^−^	*Z* = 2
*M**_r_* = 292.31	*F*(000) = 304
Triclinic, *P*1	*D*_x_ = 1.516 Mg m^−^^3^
Hall symbol: -P 1	Mo *K*α radiation, λ = 0.71069 Å
*a* = 5.818 (5) Å	Cell parameters from 4012 reflections
*b* = 9.667 (5) Å	θ = 0.4–14.1°
*c* = 11.460 (5) Å	µ = 0.27 mm^−^^1^
α = 95.241 (5)°	*T* = 293 K
β = 90.336 (5)°	Block, yellow
γ = 93.691 (5)°	0.18 × 0.15 × 0.12 mm
*V* = 640.5 (7) Å^3^	

### Data collection

**Table:**

Bruker APEXII CCD diffractometer	2572 independent reflections
Radiation source: fine-focus sealed tube	2266 reflections with *I* > 2σ(*I*)
Graphite monochromator	*R*_int_ = 0.127
φ– and ω– scans	θ_max_ = 26.4°, θ_min_ = 2.1°
Absorption correction: multi-scan (*SADABS*; Bruker, 2005)	*h* = −7→6
*T*_min_ = 0.951, *T*_max_ = 0.965	*k* = −12→11
3642 measured reflections	*l* = −14→12

### Refinement

**Table:**

Refinement on *F*^2^	Primary atom site location: structure-invariant direct methods
Least-squares matrix: full	Secondary atom site location: difference Fourier map
*R*[*F*^2^ > 2σ(*F*^2^)] = 0.066	Hydrogen site location: inferred from neighbouring sites
*wR*(*F*^2^) = 0.185	H-atom parameters constrained
*S* = 1.07	*w* = 1/[σ^2^(*F*_o_^2^) + (0.1118*P*)^2^ + 0.2488*P*] where *P* = (*F*_o_^2^ + 2*F*_c_^2^)/3
2572 reflections	(Δ/σ)_max_ < 0.001
191 parameters	Δρ_max_ = 0.54 e Å^−^^3^
0 restraints	Δρ_min_ = −0.72 e Å^−^^3^

### Special details

**Table:**

Geometry. All esds (except the esd in the dihedral angle between two l.s. planes) are estimated using the full covariance matrix. The cell esds are taken into account individually in the estimation of esds in distances, angles and torsion angles; correlations between esds in cell parameters are only used when they are defined by crystal symmetry. An approximate (isotropic) treatment of cell esds is used for estimating esds involving l.s. planes.
Refinement. Refinement of F^2^ against ALL reflections. The weighted R-factor wR and goodness of fit S are based on F^2^, conventional R-factors R are based on F, with F set to zero for negative F^2^. The threshold expression of F^2^ > 2sigma(F^2^) is used only for calculating R-factors(gt) etc. and is not relevant to the choice of reflections for refinement. R-factors based on F^2^ are statistically about twice as large as those based on F, and R- factors based on ALL data will be even larger.

### Fractional atomic coordinates and isotropic or equivalent isotropic displacement parameters (Å^2^)

**Table:**

	*x*	*y*	*z*	*U*_iso_*/*U*_eq_	Occ. (<1)
N1	0.3313 (3)	0.1672 (2)	0.84021 (18)	0.0391 (5)	
H101	0.2116	0.2132	0.8318	0.047*	
N2	0.7148 (3)	0.0191 (2)	0.86741 (19)	0.0432 (5)	
C1	0.4340 (4)	0.3195 (3)	1.0121 (2)	0.0447 (6)	
H100	0.3031	0.3686	1.0053	0.054*	
C2	0.5880 (5)	0.3558 (3)	1.1006 (2)	0.0512 (6)	
H2	0.5613	0.4302	1.1550	0.061*	
C3	0.7895 (5)	0.2821 (3)	1.1116 (2)	0.0508 (6)	
H3	0.8952	0.3112	1.1714	0.061*	
C4	0.8303 (4)	0.1705 (3)	1.0367 (2)	0.0461 (6)	
H4	0.9602	0.1214	1.0468	0.055*	
C5	0.6737 (4)	0.1283 (2)	0.9423 (2)	0.0380 (5)	
C6	0.4758 (4)	0.2065 (2)	0.9312 (2)	0.0372 (5)	
C7	0.3673 (4)	0.0595 (3)	0.7627 (2)	0.0406 (5)	
C8	0.2129 (5)	0.0209 (3)	0.6681 (2)	0.0538 (7)	
H8	0.0821	0.0694	0.6588	0.065*	
C9	0.2593 (6)	−0.0875 (3)	0.5918 (3)	0.0640 (8)	
H9	0.1585	−0.1136	0.5292	0.077*	
C10	0.4572 (6)	−0.1629 (3)	0.6041 (3)	0.0636 (8)	
H10	0.4855	−0.2361	0.5487	0.076*	
C11	0.6058 (5)	−0.1301 (3)	0.6951 (3)	0.0557 (7)	
H11	0.7332	−0.1820	0.7032	0.067*	
C12	0.5663 (4)	−0.0160 (3)	0.7782 (2)	0.0414 (5)	
S1	0.88381 (9)	0.37619 (6)	0.71184 (5)	0.0422 (3)	
O1A	0.8047 (4)	0.5126 (3)	0.7145 (2)	0.0583 (8)	0.853 (5)
O2	1.0304 (4)	0.3550 (2)	0.81033 (17)	0.0602 (6)	
O3	0.7187 (5)	0.2637 (3)	0.6816 (3)	0.0894 (9)	
O4A	1.0400 (4)	0.3553 (3)	0.59643 (19)	0.0545 (7)	0.853 (5)
C13	1.2319 (5)	0.4504 (4)	0.5904 (3)	0.0679 (9)	
H13A	1.1875	0.5429	0.6129	0.102*	
H13B	1.2884	0.4454	0.5117	0.102*	
H13C	1.3507	0.4278	0.6426	0.102*	
O1B	1.035 (3)	0.4806 (16)	0.6513 (14)	0.063 (5)*	0.147 (5)
O4B	0.718 (3)	0.4697 (18)	0.7705 (17)	0.069 (5)*	0.147 (5)

### Atomic displacement parameters (Å^2^)

**Table:**

	*U*^11^	*U*^22^	*U*^33^	*U*^12^	*U*^13^	*U*^23^
N1	0.0301 (9)	0.0466 (11)	0.0416 (10)	0.0090 (8)	−0.0058 (8)	0.0053 (8)
N2	0.0344 (10)	0.0489 (12)	0.0471 (11)	0.0108 (8)	−0.0026 (9)	0.0032 (9)
C1	0.0416 (13)	0.0469 (13)	0.0465 (13)	0.0125 (10)	−0.0029 (11)	0.0034 (10)
C2	0.0561 (15)	0.0510 (14)	0.0457 (14)	0.0085 (12)	−0.0037 (12)	−0.0027 (11)
C3	0.0452 (14)	0.0609 (16)	0.0455 (13)	0.0025 (12)	−0.0137 (11)	0.0018 (11)
C4	0.0337 (12)	0.0571 (15)	0.0487 (13)	0.0088 (10)	−0.0081 (11)	0.0077 (11)
C5	0.0285 (10)	0.0443 (12)	0.0420 (12)	0.0061 (9)	−0.0010 (9)	0.0063 (9)
C6	0.0313 (11)	0.0426 (12)	0.0389 (11)	0.0054 (9)	−0.0021 (9)	0.0073 (9)
C7	0.0347 (11)	0.0465 (13)	0.0406 (12)	0.0027 (9)	−0.0030 (10)	0.0041 (10)
C8	0.0491 (15)	0.0617 (16)	0.0497 (14)	0.0034 (12)	−0.0147 (12)	0.0017 (12)
C9	0.0687 (19)	0.0686 (19)	0.0517 (16)	0.0006 (15)	−0.0150 (14)	−0.0065 (14)
C10	0.072 (2)	0.0604 (17)	0.0547 (16)	0.0045 (15)	0.0017 (15)	−0.0138 (13)
C11	0.0527 (15)	0.0530 (15)	0.0603 (16)	0.0112 (12)	0.0039 (13)	−0.0057 (12)
C12	0.0346 (11)	0.0471 (13)	0.0424 (12)	0.0040 (9)	0.0013 (10)	0.0029 (10)
S1	0.0289 (3)	0.0536 (4)	0.0452 (4)	0.0116 (2)	−0.0043 (2)	0.0049 (3)
O1A	0.0508 (14)	0.0665 (16)	0.0608 (15)	0.0302 (12)	−0.0018 (12)	0.0050 (12)
O2	0.0572 (12)	0.0763 (14)	0.0491 (11)	0.0274 (10)	−0.0149 (9)	0.0008 (9)
O3	0.0926 (19)	0.0844 (18)	0.0879 (18)	−0.0211 (15)	−0.0266 (16)	0.0104 (14)
O4A	0.0513 (13)	0.0639 (15)	0.0479 (13)	0.0122 (11)	0.0020 (10)	−0.0033 (10)
C13	0.0365 (13)	0.107 (3)	0.0619 (17)	0.0065 (15)	0.0059 (13)	0.0143 (17)

### Geometric parameters (Å, º)

**Table:**

N1—C7	1.333 (3)	C8—H8	0.9300
N1—C6	1.347 (3)	C9—C10	1.415 (5)
N1—H101	0.8600	C9—H9	0.9300
N2—C5	1.333 (3)	C10—C11	1.353 (4)
N2—C12	1.340 (3)	C10—H10	0.9300
C1—C2	1.355 (4)	C11—C12	1.422 (4)
C1—C6	1.401 (3)	C11—H11	0.9300
C1—H100	0.9300	S1—O3	1.421 (3)
C2—C3	1.421 (4)	S1—O1A	1.423 (2)
C2—H2	0.9300	S1—O2	1.448 (2)
C3—C4	1.350 (4)	S1—O4B	1.486 (18)
C3—H3	0.9300	S1—O1B	1.516 (16)
C4—C5	1.424 (3)	S1—O4A	1.614 (2)
C4—H4	0.9300	O4A—C13	1.406 (4)
C5—C6	1.429 (3)	C13—H13A	0.9600
C7—C8	1.412 (3)	C13—H13B	0.9600
C7—C12	1.426 (4)	C13—H13C	0.9600
C8—C9	1.345 (4)		
			
C7—N1—C6	122.4 (2)	C8—C9—C10	121.8 (3)
C7—N1—H101	118.8	C8—C9—H9	119.1
C6—N1—H101	118.8	C10—C9—H9	119.1
C5—N2—C12	118.3 (2)	C11—C10—C9	121.1 (3)
C2—C1—C6	118.8 (2)	C11—C10—H10	119.5
C2—C1—H100	120.6	C9—C10—H10	119.5
C6—C1—H100	120.6	C10—C11—C12	119.6 (3)
C1—C2—C3	121.2 (2)	C10—C11—H11	120.2
C1—C2—H2	119.4	C12—C11—H11	120.2
C3—C2—H2	119.4	N2—C12—C11	120.2 (2)
C4—C3—C2	121.2 (2)	N2—C12—C7	121.7 (2)
C4—C3—H3	119.4	C11—C12—C7	118.1 (2)
C2—C3—H3	119.4	O3—S1—O1A	116.76 (18)
C3—C4—C5	119.7 (2)	O3—S1—O2	113.70 (16)
C3—C4—H4	120.1	O1A—S1—O2	114.03 (13)
C5—C4—H4	120.1	O3—S1—O4B	95.5 (7)
N2—C5—C4	119.9 (2)	O1A—S1—O4B	37.2 (7)
N2—C5—C6	122.1 (2)	O2—S1—O4B	100.4 (7)
C4—C5—C6	118.0 (2)	O3—S1—O1B	138.7 (6)
N1—C6—C1	121.5 (2)	O1A—S1—O1B	64.2 (6)
N1—C6—C5	117.4 (2)	O2—S1—O1B	100.5 (6)
C1—C6—C5	121.1 (2)	O4B—S1—O1B	100.4 (10)
N1—C7—C8	121.1 (2)	O3—S1—O4A	97.18 (17)
N1—C7—C12	118.1 (2)	O1A—S1—O4A	106.51 (15)
C8—C7—C12	120.9 (2)	O2—S1—O4A	106.33 (13)
C9—C8—C7	118.5 (3)	O4B—S1—O4A	142.5 (8)
C9—C8—H8	120.7	O1B—S1—O4A	49.6 (6)
C7—C8—H8	120.7	C13—O4A—S1	116.1 (2)
			
C6—C1—C2—C3	0.4 (4)	C12—C7—C8—C9	−0.9 (4)
C1—C2—C3—C4	−2.3 (5)	C7—C8—C9—C10	0.0 (5)
C2—C3—C4—C5	2.3 (4)	C8—C9—C10—C11	1.3 (6)
C12—N2—C5—C4	−178.6 (2)	C9—C10—C11—C12	−1.6 (5)
C12—N2—C5—C6	1.0 (4)	C5—N2—C12—C11	178.9 (2)
C3—C4—C5—N2	179.2 (2)	C5—N2—C12—C7	−0.5 (4)
C3—C4—C5—C6	−0.5 (4)	C10—C11—C12—N2	−178.7 (3)
C7—N1—C6—C1	−179.6 (2)	C10—C11—C12—C7	0.7 (4)
C7—N1—C6—C5	0.3 (4)	N1—C7—C12—N2	−0.1 (4)
C2—C1—C6—N1	−178.8 (2)	C8—C7—C12—N2	180.0 (2)
C2—C1—C6—C5	1.4 (4)	N1—C7—C12—C11	−179.5 (2)
N2—C5—C6—N1	−0.9 (4)	C8—C7—C12—C11	0.6 (4)
C4—C5—C6—N1	178.8 (2)	O3—S1—O4A—C13	179.8 (2)
N2—C5—C6—C1	178.9 (2)	O1A—S1—O4A—C13	59.1 (3)
C4—C5—C6—C1	−1.4 (4)	O2—S1—O4A—C13	−62.9 (3)
C6—N1—C7—C8	−179.9 (2)	O4B—S1—O4A—C13	70.9 (11)
C6—N1—C7—C12	0.2 (4)	O1B—S1—O4A—C13	27.2 (8)
N1—C7—C8—C9	179.2 (3)		

### Hydrogen-bond geometry (Å, º)

**Table:**

*D*—H···*A*	*D*—H	H···*A*	*D*···*A*	*D*—H···*A*
N1—H101···O2^i^	0.86	1.82	2.647 (5)	161

Symmetry code: (i) *x*−1, *y*, *z*.
